# Chia Seed Oil Ameliorates Doxorubicin-Induced Cardiotoxicity in Female Wistar Rats: An Electrocardiographic, Biochemical and Histopathological Approach

**DOI:** 10.1007/s12012-021-09644-3

**Published:** 2021-03-19

**Authors:** Akheruz Zaman Ahmed, Kamalesh D. Mumbrekar, Shakta Mani Satyam, Prakashchandra Shetty, Melanie Rose D’Souza, Varun Kumar Singh

**Affiliations:** 1 Department of Anatomy, Melaka Manipal Medical College, Manipal Academy of Higher Education, Manipal, Karnataka India; 2 Department of Radiation Biology &Toxicology, Manipal School of Life Sciences, Manipal Academy of Higher Education, Manipal, Karnataka India; 3 Department of Pharmacology, Melaka Manipal Medical College, Manipal Academy of Higher Education, Manipal, Karnataka India; 4 Department of Pathology, Melaka Manipal Medical College, Manipal Academy of Higher Education, Manipal, Karnataka India

**Keywords:** Anthracyclines, Chemotherapy, Acute cardiotoxicity, Electrocardiogram (ECG), Nutraceuticals, Antioxidants

## Abstract

Doxorubicin (DOX) is a potent anti-cancer antibiotic that was widely used for treatment of various cancers. It produces free radicals which result in extreme dose-limiting cardiotoxicity. This study investigated the cardioprotective potential of chia seed oil, an active polyphenolic nutraceutical against doxorubicin-induced cardiotoxicity in Wistar rats. Twenty-four female Wistar rats were divided into four groups (*n* = 6) which consist of normal control, DOX control, test-A and test-B group. Animals were prophylactically treated with two different doses of test drug, i.e. chia seed oil 2.5 ml/kg/day and 5 ml/kg/day in test-A and test-B groups orally for 7 days. Doxorubicin (25 mg/kg; single dose) was administered intraperitoneally to DOX control, Test-A and Test-B animals on the seventh day to induce cardiotoxicity. ECG analysis was done before and after treatment. Besides ECG, CK, CK-MB, LDH, AST, MDA and GSH were analyzed. DOX had significantly altered ECG, CK, CK-MB, LDH, AST, MDA and GSH. Pre-treatment with chia seed oil significantly alleviated DOX-induced ECG changes and also guarded against DOX-induced rise of serum CK, CK-MB and AST levels. Chia seed oil alleviated histopathological alteration in DOX-treated rats. It also significantly inhibited DOX-induced GSH depletion and elevation of MDA. The present study revealed that chia seed oil exerts cardioprotection against doxorubicin-induced cardiotoxicity in female Wistar rats. Our study opens the perspective to clinical studies to precisely consider chia seed oil as a potential chemoprotectant nutraceutical in the combination chemotherapy with doxorubicin to limit its cardiotoxicity.

## Introduction

Doxorubicin is a potent cytotoxic anticancer antibiotic separated from the culture of *Streptomyces peucetius* and used to treat different types of cancers like breast, ovarian, endometrial, bladder, thyroid, acute leukaemia, Hodgkin’s disease, Wilms tumor, multiple myeloma [1]. Although it is a potent anticancer antibiotic, toxicities including nausea, vomiting, alopecia, and hematopoietic suppression, as well as a unique cardiotoxicity induced by DOX limit the use of this antibiotic. DOX is more prone to heart tissue toxicities and constitutes a major cause of morbidity and mortality in cancer patients [2].

An imbalance between the production of ROS and RNS free radicals and their elimination by the cellular antioxidant system results in oxidative stress. ROS and RNS are generated in mitochondria from dioxygen and nitric oxide, respectively. The heart is rich with mitochondria, which constitutes approximately 40% of the total intracellular volume of cardiomyocytes [3]. Since evidence points out to a preferential interaction of DOX with cardiac mitochondria, several mitochondrial alterations have been measured in different models [4]. DOX has great affinity for the negatively charged cardiolipin, which is abundant in the mitochondrial inner membrane [5] and leads to accumulation of DOX in this organelle. It was determined that under clinically relevant plasma DOX concentrations of 0.5–1 μM can reach up to 50–100 μM in mitochondria [6]. This high concentration of the drug leads to a high redox reactivity in the heart [7].

DOX-induced reactive oxygen species (ROS) causes oxidative damage to biological macromolecules, including lipids, proteins, DNA, and affects the structure and functions of cardiac cell membranes [8]. DOX also reduces endogenous antioxidants and increases lipid peroxidation that alters the cardiac function [9]. Besides, they significantly decrease the reduced glutathione (GSH) level. In general, antioxidant resources are low in cardiomyocytes as compared to other organs in the body, making the heart more vulnerable to free radical damage by DOX [10].

In cardiovascular research, electrocardiography (ECG) is widely used to monitor cardiac dysfunction because ECG is affordable and commonly available. The electrocardiographic changes associated with cardiomyopathy caused by doxorubicin initially include multiple reversible arrhythmias, most commonly sinus tachycardia [11, 12]. Some of the electrocardiographic features observed later with prolonged cardiotoxicity of doxorubicin are associated with T-wave flattening, QT-interval prolongation, and also R wave voltage loss [13].

Antioxidants are molecules that can suppress ROS and reduce oxidative stress damage. Even though extensive researches have been done to reduce cardiotoxicity caused by DOX, no effective preventive measure has yet been discovered. Chia seed oil (CSO) is derived from Chia seeds (Salvia hispanica L.) that are used traditionally for medicinal properties [14, 15]. CSO contains high amount of polyunsaturated fatty acids (PUFA), mainly α-linolenic acid (omega -3 fatty acids), oleic acids and palmitic acids [16]. Preclinical and clinical studies have verified that omega -3 fatty acids possess anti-inflammatory, antiarrhythmic, antithrombotic, neuroprotective, anticancer, antidepressant and immunomodulatory activities, making it an important class of drug and adjunctive therapeutics for disease therapy [17, 18].

The cardioprotective potential of chia seed oils has not been reported yet. Hence the present study aims to investigate the cardioprotective potential of chia seed oil against DOX-induced cardiotoxicity in female Wistar rats.

## Materials and Methods

### Animals

A total of 24 adult female Wistar rats (age: 8–10 weeks old & body weight: 150–200 g) bred in Central Animal House, Manipal, Manipal Academy of Higher Education (MAHE) were housed in separate polypropylene cages. Animals were kept at temperatures (22–24 °C), 12-h light/12-h dark cycle and 40%-60% relative air humidity under standard conditions. Rats had continuous access to tap water with regular rat pellet diet on normal calories (Hindustan Lever Ltd., Mumbai, India). After randomization into various study groups, the rats were acclimatised for one week before starting the experiment under the same laboratory conditions. The experimental protocol was approved by the Institutional Animal Ethics Committee (IAEC/KMC/113/2019), and experiments were conducted in accordance with the ethical standards approved by the Ministry of Social Justice and Empowerment (Government of India) and the guidelines of CPCSEA.

### Chemicals

Doxorubicin was purchased from Cipla Ltd., Goa (India). Assay kits for creatine kinase-MB (CK-MB), creatine kinase (CK), lactate dehydrogenase (LDH) and aminotransferase (AST) were obtained from ASPEN Laboratories, New Delhi (India). Thiobarbituric acid (TBA), trichloroacetic acid (TCA), 5,5′-dithiobis (2-nitrobenzoic acid) (DTNB) and reagents for histopathological analysis were procured from Sigma Aldrich-Merck, Bangalore (India). All reagents were analytical grade. Before the biochemical estimations, reagents were stabilized at room temperature for 30 min.

### Extraction of Chia Seed Oil

Extraction of oil from black Chia Seed was done at Bargi Naturals, Mysore, India using cold press extraction methods [19]. The oil was permitted to stand for a sedimentation time of 1 week after extraction to eliminate solid impurities. The oil was then filtered and placed in sealed dark coloured bottles protected by aluminium foils below 4° C.

### Animal Selection and Grouping

Baseline screening for any cardiac abnormality was done through BPL cardiart from the lead II ECG (BPL-91018, India) of all the experimental animals. Animals showing depressed ST segment/absence of P-wave/inverted P-wave/non-specific ST segment/ST segment elevation were excluded from the experiment. A total of 24 rats showing normal ECG were included in the study and divided into four groups (*n* = 6) and treated as follows:

#### Group I

(Vehicle control): 2% Dimethyl sulfoxide (DMSO) in double-distilled water; 1 ml/kg/day orally for 7 days + 0.9% NaCl, 1 ml/kg (single dose); i.p. on 7th day.

#### Group II

(DOX control): DOX 25 mg/kg (single dose); i.p. on 7th day.

#### Group III

(Test A; DOX + CSO 2.5 ml/kg): CSO 2.5 ml/kg/day orally for 7 days + DOX 25 mg/kg (single dose); i.p. on 7th day.

#### Group IV

(Test B; DOX + CSO 5 ml/kg): CSO 5 ml/kg/day orally for 7 days + DOX 25 mg/kg (single dose); i.p. on 7th day.

On the 8th day (24 h after the administration of DOX), all the experimental animals were anaesthetized by intraperitoneal administration of both ketamine (60 mg/kg) and xylazine (10 mg/kg). The widely used therapeutic dose of doxorubicin is 60–75 mg/m2 IV once every 21 days to treat varieties of cancers. This dose is equivalent to 20–25 mg/kg in rats [20]. Two doses of chia seed oil (2.5 ml/kg and 5 ml/kg) were selected based on our results of the acute toxicity study (ATC method; OECD 423 guideline) for chia seed oil.

### ECG Recording

Following anaesthesia, each rat was positioned on the animal operation table for ECG recording. Electrodes were tied on palmer surface of clean-shaven limbs of the rat. The front limbs and left hind limb were used for the recording of ECG in standard leads, while the right hind limb was attached with grounded electrode. A conductive ECG gel was applied with care over each electrode to prevent a gel bridge between them from being formed. ECG was recorded for each animal for one minute and averages of data from 11 consecutive ECG signals were analysed quantitatively in terms of PR interval, QT interval, QTc interval and QRS complex amplitude. In addition to these ST segment was also analysed qualitatively.

### Collection of Blood and Serum Preparation

Anaesthetized animals were euthanized after the blood collection. The heart was collected from the mediastinum by dissecting it out from the major blood vessels. Gross examination of the heart was done to check MI as per the criteria given by Wu et al. [21]. The heart was then washed in regular saline, soaked on blotting paper to extract the blood and then set for histopathological analysis in 10% formalin.

### Estimation of LDH, CK, CK-MB and AST

LDH, CK, CK-MB and AST were measured using a semiautoanalyzer (Star 21 Plus, Mumbai, India) as per the standard protocol given along with the respective commercially available kits.

### Estimation of Malondialdehyde (MDA) & Reduced Glutathione (GSH)

Serum was analyzed for both MDA and GSH as per the protocol given by Satyam et al. [22]. Optical density was read at 540 nm and 412 nm for MDA and GSH, respectively, using iMark microplate absorbance reader (Bio-Rad Laboratories, USA). Serum MDA and GSH levels were calculated based on their absorbance and both were expressed as mM/ml.

### Histopathological Analysis

After 24 h of fixation of heart tissues in 10% formalin, tissue samples were dehydrated in ascending concentrations of ethanol, cleared in xylene and embedded in paraffin to prepare the block. Then, histological sections of 5 μm thicknesses were taken using rotary microtome and staining was done using Haematoxylin & Eosin (H & E). Inter muscular edema, myofibrillar loss, infiltration with inflammatory cells, vacuolization & cardiomyocytes degeneration were assessed from all the experimental groups. All the pathological findings were verified by a pathologist.

### Statistical Analysis

Using the Statistical Package for the Social Sciences (SPSS version 16.0; SPSS), data expressed as mean ± standard deviation and analyzed by one-way analysis of variance (ANOVA) followed by post hoc Tukey test. A level for *p* ≤ 0.05 was considered to be statistically significant.

## Results

### Effect on Electrocardiography

**Fig. 1:**
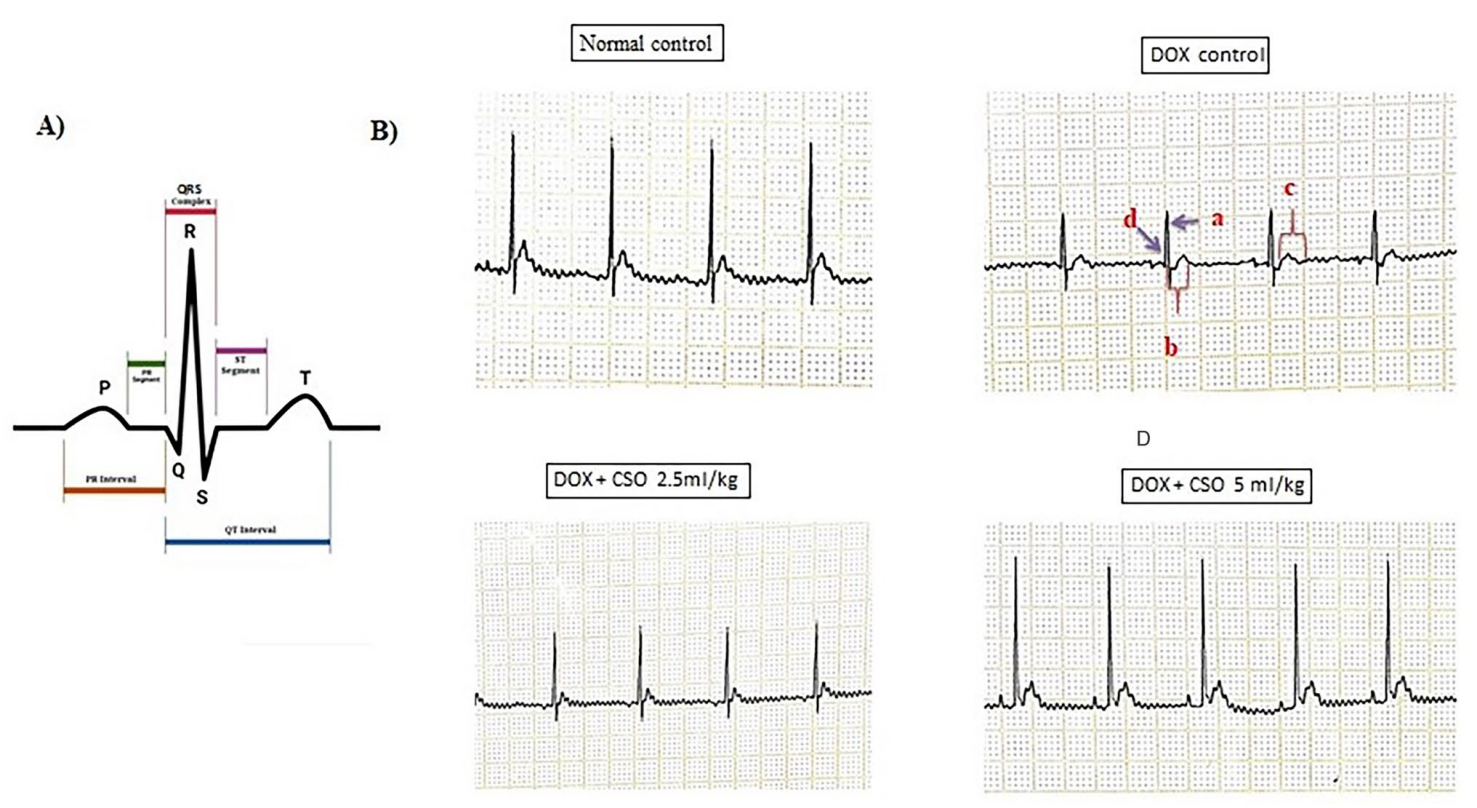
**A** Typical ECG graph showing PR interval, QRS complex, ST segment, QT interval. **B** Representative ECG of normal control, DOX control, DOX + CSO 2.5 ml/kg and DOX + CSO 5 ml/kg. **a** Reduced QRS complex amplitude, **b** Prolongation of QT interval, **c** Non-specific ST segment, **d** Increased PR interval

QT interval prolongation, reduction in QRS complex and ST segment changes are some of the characteristic ECG findings in DOX-induced cardiomyopathy. In DOX-treated animals, increased PR interval, prolongation of QT & QTc interval, reduced QRS complex amplitude and non-specific ST segment were observed. However, these changes were not seen in vehicle control and test groups (Fig. 1).

**Fig. 2:**
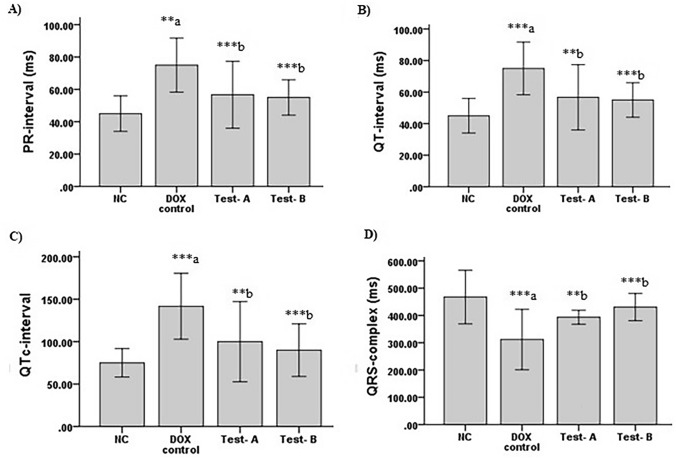
Effect of DOX and CSO on ECG parameters. **A** PR interval (ms), B QT interval (ms), **C** QTc-interval (ms), **D** QRS complex (ms). Data were represented as mean ± SD, and one-way ANOVA followed by Tukey’s post hoc test (***p ≤ 0.001, **p ≤ 0.01, *p ≤ 0.05 ^a^ compared to control, ^b^ compared to DOX control), NC: Normal control, Test-A: DOX + CSO 2.5 ml/kg and Test-B: DOX + CSO 5 ml/kg

There was a significant increase in PR interval (p = 0.003), QT interval (*p* < 0.001), QTc interval (*p* < 0.001) and decrease in QRS complex amplitude (*p* < 0.001) among DOX control animals in comparison with normal control animals. PR interval (*p* < 0.001), QT interval (*p* = 0.003), QTc interval (*p* = 0.003) were significantly decreased and QRS complex amplitude (*p* = 0.010) was increased among Test-A (DOX + CSO 2.5 ml/kg) treated group compared to DOX control group. A significant decrease was also observed in PR interval (*p* < 0.001), QT interval (*p* < 0.001), QTc interval (*p* < 0.001) and increase in QRS complex amplitude (*p* < 0.001) in Test-B (DOX + CSO 5 ml/kg) group in comparison with DOX control animals (Fig. 2).

### Amelioration of Serum CK-MB, CK, LDH and AST Activity

**Fig. 3:**
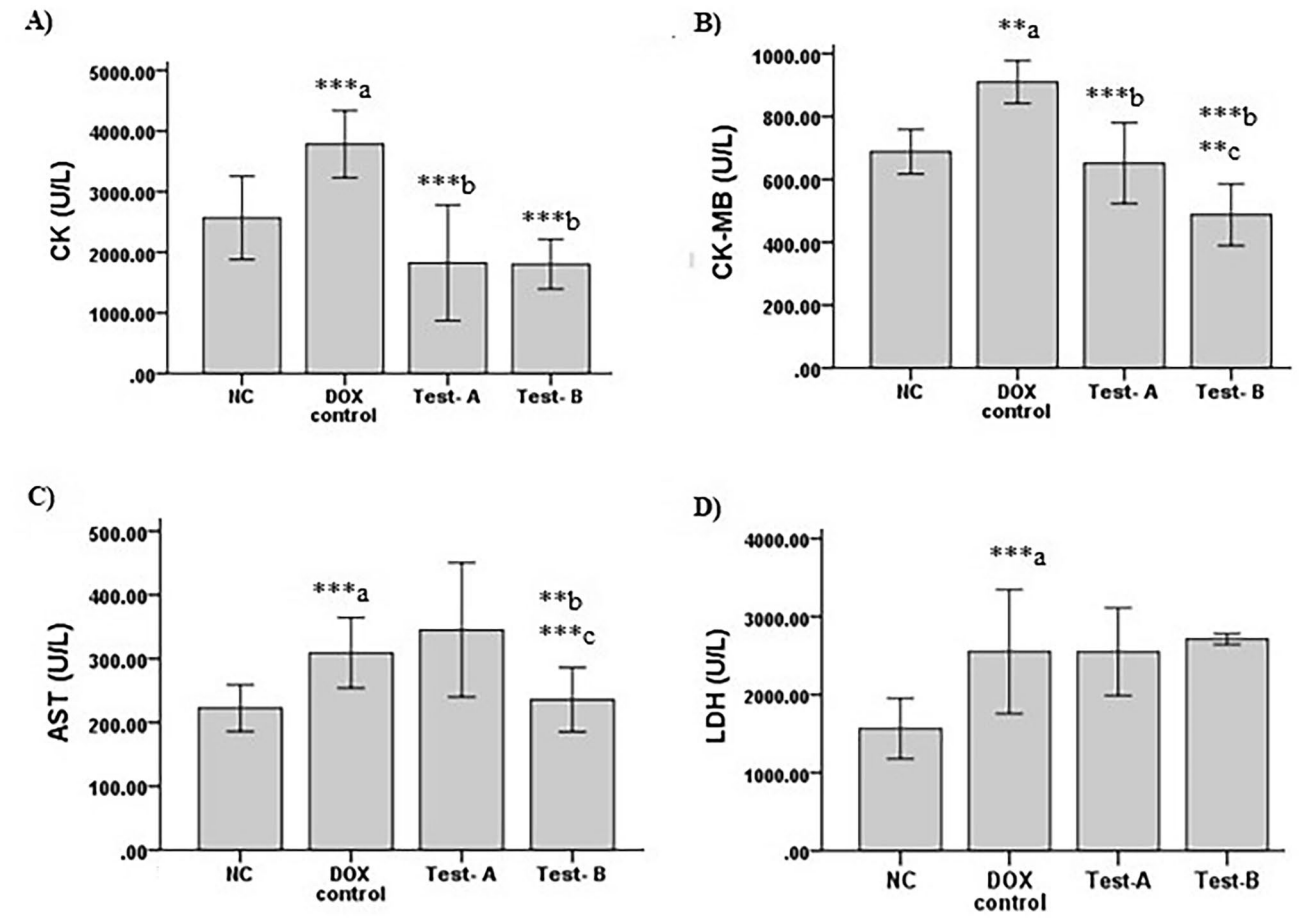
Effect of DOX and CSO on serum cardiac injury biomarkers. **A** Creatinine kinase. **B** Creatinine kinase-MB. **C** Aspartate aminotransferase. **D** Lactate dehydrogenase. Data were represented as mean ± SD, one-way ANOVA followed by Tukey’s post hoc test (***p ≤ 0.001, **p ≤ 0.01, *p ≤ 0.05 ^a^ compared to control, ^b^ compared to DOX control), NC: Normal control, Test-A: DOX + CSO 2.5 ml/kg and Test-B: DOX + CSO 5 ml/kg

Measurements of the serum cardiac markers like CK-MB, CK, LDH and AST were done to understand cardiotoxicity induced by DOX and the protective effect of CSO. There was a significant increase in CK-MB (*p* < 0.01), CK (*p* < 0.001), LDH (*p* < 0.001) and AST (*p* < 0.001) in DOX control group in comparison with normal control. A significant decrease was observed for CK-MB (*p* < 0.001) and CK (*p* < 0.001) in the Test-A group. Similarly in Test-B group also a decrease in CK-MB (*p* < 0.001), CK (*p* < 0.001) and AST (*p* < 0.01) was observed as compared to DOX control group. CSO 5 ml/kg has significantly reduced CK-MB (*p* < 0.001) compared to CSO 2.5 ml/kg treated group (Fig. 3). The increase in CK-MB, CK, LDH and AST indicates cellular damage and loss of functional integrity of cardiomyocytes. These findings had shown cardiotoxicity by DOX in female Wistar rats. Treatment with CSO ameliorated increase AQ levels of CK-MB, CK and AST, indicating its cardioprotective effect against DOX.

### Effect on Serum GSH and MDA Levels

**Fig. 4:**
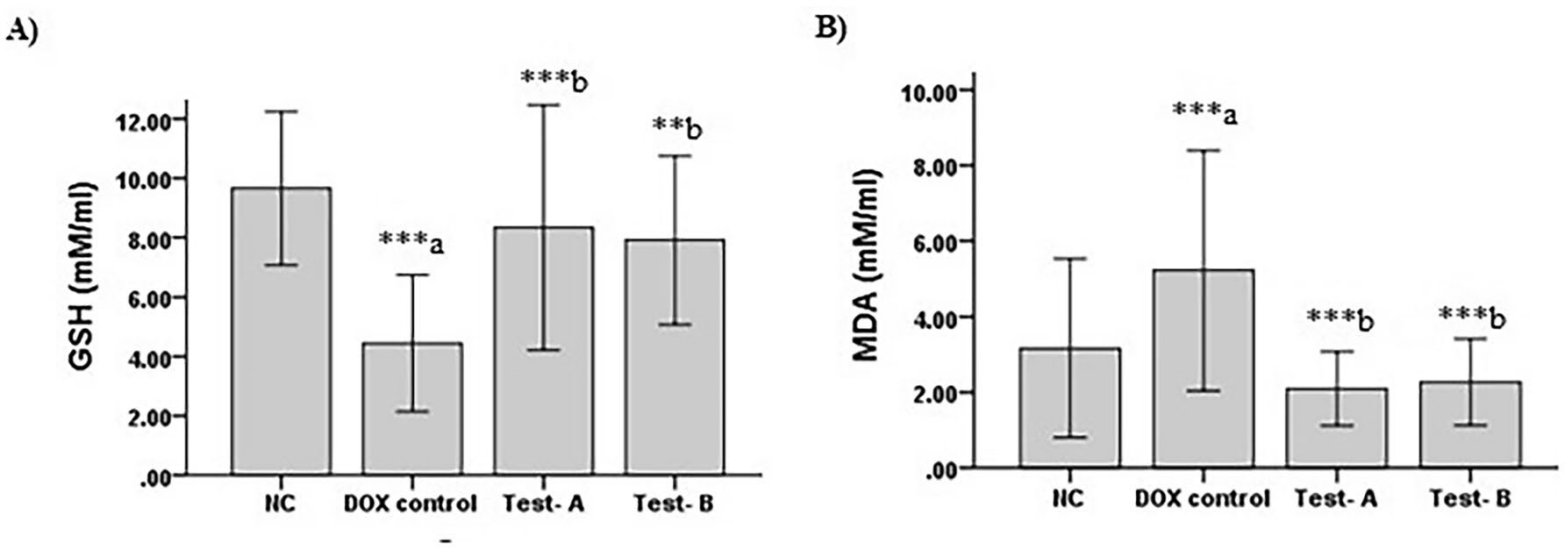
Effect of DOX and CSO on Oxidative stress parameters. **A** Reduced glutathione. **B** Malondialdehyde**.**. Data were represented as mean ± SD, one-way ANOVA followed by Tukey’s post hoc test (***p ≤ 0.001, **p ≤ 0.01, *p ≤ 0.05 ^a^ compared to control, ^b^ compared to DOX control), NC: Normal control, Test-A: DOX + CSO 2.5 ml/kg and Test-B: DOX + CSO 5 ml/kg

In the DOX control group, there was a significant decrease in GSH (*p* < 0.001) whereas MDA (*p* = 0.015) was significantly increased compared to normal control animals. CSO 2.5 ml/kg (Test-A) has significantly increased GSH (*p* < 0.001) and decreased MDA (*p* < 0.001) levels in comparison with the rats treated with the DOX control group. Similarly, there was a significant increase in GSH (*p* = 0.004) and decrease in MDA (*p* < 0.001) among the rats treated with CSO 5 ml/kg compared to the DOX control group (Fig. 4).

### Gross Examination of Isolated Hearts

**Fig. 5:**
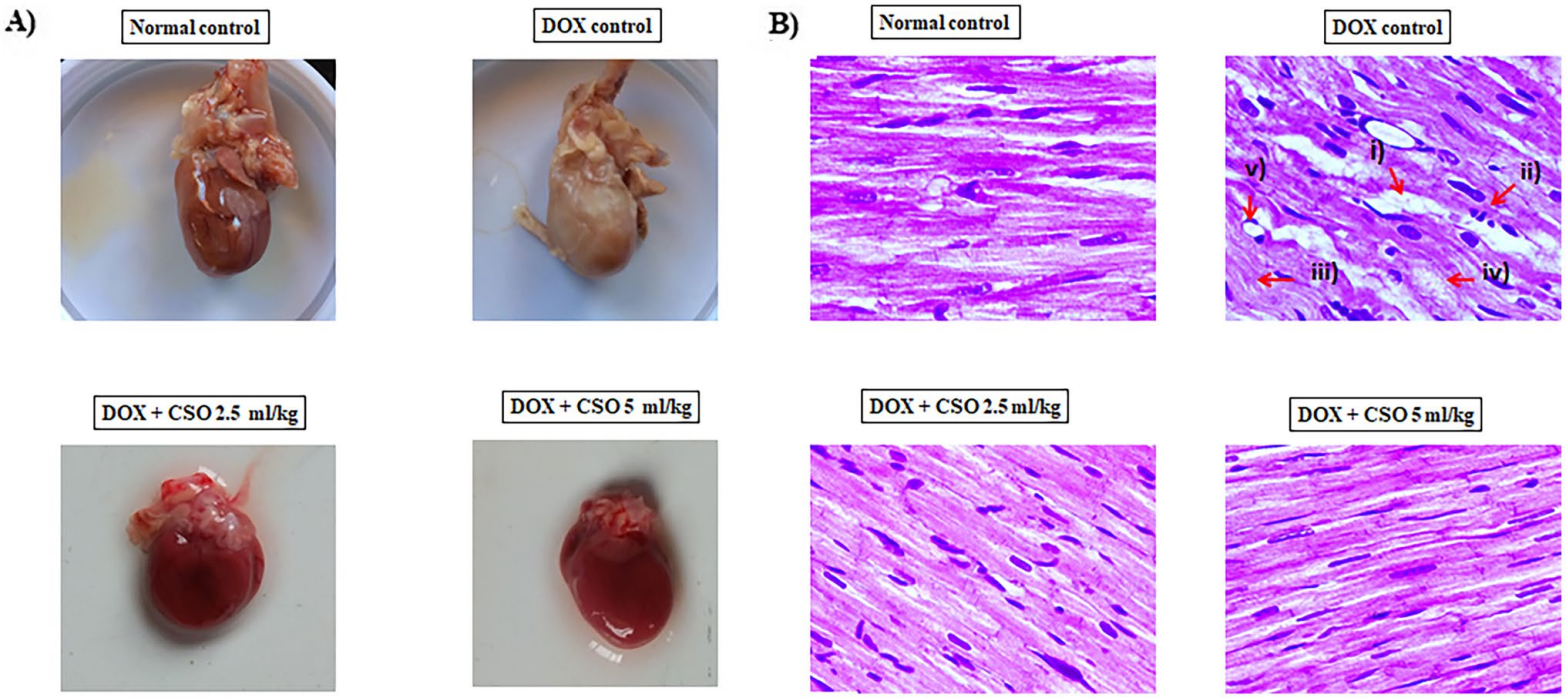
Effect of DOX and CSO on heart anatomy. **A** Representative photographs of isolated hearts after the end of the experiment. **B** Longitudinal section of cardiac tissue stained with H & E under 400X, ( i Inter muscular edema, ii Infiltration with inflammatory cells, iii Myofibrillar loss, iv Cardiomyocytes degeneration, v Vacuolization)

Myocardial infarction (MI) was commonly noted among isolated hearts of DOX control rats. MI as pale/yellow with hyperemic or hemorrhagic borders/white–grey (scar) was significantly noted among isolated hearts of DOX control rats, whereas these were absent in normal control and test groups (Fig. 5a).

### Histopathological Examination of Cardiac Tissue

DOX-induced cardiomyopathy was further examined by using H&E staining under a light microscope. The normal control group had shown typical cardiomyocytes architecture. In DOX-treated control group’s cardiomyocytes had presented intermuscular edema, myofibrillar loss, infiltration with inflammatory cells, vacuolization and cardiomyocytes degeneration. All these pathological changes were mitigated among test-A and test-B group and cardiomyocytes architecture almost looked like normal control group (Fig. 5b).

## Discussion

DOX-induced cardiotoxicity is known to have an extremely severe adverse effect of oncology therapy. After its discovery, multiple molecular mechanisms have been suggested to explain the pathogenesis of acute and chronic cardiotoxicity caused by DOX, including oxidative stress, iron metabolism, Ca2 + homeostasis dysregulation, gene expression modulation, sarcomeric structure alterations and apoptosis [23]. Different methods to protect the heart during cancer treatment have been developed based on these mechanisms. Chia seed is an annual herbaceous plant and rich in antioxidants. This study evaluated the cardioprotective potential of chia seed oil against DOX-induced cardiotoxicity in female Wistar rats. The results of this study showed cardioprotective potential of chia seed oil in female Wistar rats against DOX-induced cardiotoxicity. DOX is more commonly used in the treatment of breast cancer. Hence female Wistar rats were selected in this study.

Acute cardiotoxicity caused by DOX is observed during and within 2–3 days of its single dose of DOX administration [24, 25]. The present study has observed acute cardiotoxicity with a single dose of DOX administration to the experimental animals. DOX has significantly altered ECG waves in the form of increased PR interval, prolonged QT and QTc interval, reduced QRS complex amplitude and nonspecific ST segment. The findings of all these ECG changes in the present study were supported by previously reported studies [26–28]. Due to DOX-induced lipid peroxidation, an altered membrane structure may be responsible for most ECG changes. Both the doses of chia seed oils (2.5 ml/kg and 5 ml/kg) have significantly mitigated these acute changes in ECG.

The present study had observed significantly high serum LDH, CK, CK-MB and AST in DOX control group as compared to normal control group. Previously, studies had reported high serum LDH, CK, CK-MB and AST levels in rats treated with DOX that is corroborated with the finding of the present study:, serum cardiac biomarkers are released from damaged cardiomyocytes and are sensitive indicators of DOX-induced cardiotoxicity [29, 30]. CSO had significantly inhibited DOX-induced elevation of serum CK, CK-MB and AST levels. These biochemical data and ECG abnormalities were further supported by histopathological examination of cardiomyocytes. The histopathological examination of cardiac tissue had shown cardiac injury in the form of intermuscular edema, myofibrillar loss, infiltration with inflammatory cells, vacuolization and cardiomyocytes degeneration. These histological findings are linked with the previously published data [19, 30]. Pre-treatment with CSO had reduced DOX-induced histological alteration of cardiomyocytes as compared to those only treated with DOX. The observed protection of cardiomyocytes integrity might lead to decreased leakage of cardiac bio-markers in serum that was observed in this study.

Oxidative stress is a keystone in DOX-induced cardiotoxicity [31], hence this present study also investigated the non-enzymatic antioxidant status of the animals. The data had shown significantly high MDA in DOX control group as compared to normal control group and GSH was significantly reduced following DOX administration. These data clearly show that there is overt oxidative stress. The findings of the present study data are linked with previous investigations [32, 33]. The deficiency of GSH and the increase in MDA level produced by DOX may be attributed to the depletion of GSH in the interactions of DOX-induced free radicals with biomembrane and following lipid peroxidation. Pre-treatment with CSO significantly protects the animals against the DOX-induced oxidative stress. Our results had shown that CSO improved the antioxidant activities in rats that were decreased by DOX. This might be on the basis of its high content of numerous antioxidants including α-linolenic acid and polyphenols [16]. Because of its potent anti-oxidant properties, CSO were reported to inhibit oxidative induced damages [34, 35]. Many studies have reported that α-linolenic acid possess potent anti-inflammatory, anti-oxidant, antiarrhythmic, antithrombotic, neuroprotective, anticancer, antidepressant and immunomodulatory properties [17, 18].

ROS is a small biological molecule that is continuously synthesized as a natural end product of oxygen metabolism in aerobic organisms. In cell signalling, ROS plays a significant physiological function, while long-term cell exposure to elevated ROS levels will produce profound toxic effects, such as apoptotic cell death and necrotic [36]. Non-enzymatic synthesis of ROS occurs primarily in mitochondria, especially in the mitochondrial electron transport chain complexes I and III [37]. Oxidative stress is defined by the deleterious processes induced by an imbalance between the formation of ROS and minimal antioxidant defences. Disturbance of the cellular redox balance, due to increased ROS synthesis and limited antioxidant defences, contributes to oxidative changes of biological macromolecules such as nucleic acids, lipids and proteins [38]. Particularly, mitochondrial DNA is highly vulnerable to ROS-induced damages because it is located in close proximity to the synthesis site of ROS and mitochondrial DNA repair mechanisms are hampered. As a consequence, mitochondrial DNA deletions accumulate, eventually leading to a decrease in mitochondrial function and concomitant increased production of ROS and cell becomes more prone to death [39].

ROS is produced by one of the mechanisms of DOX to induce toxicity [20]. One of the studies reported that cardiomyocytes contain highest number of mitochondria which are the main source for ROS production [3]. Antioxidants are the keys of defence mechanisms to scavenge ROS to protect the host against damages [40]. With its rich antioxidant properties, CHO has the ability to scavenge ROS produced by DOX and protect the tissue from oxidative stress damages. Many studies reported that pre-treatment with omega-3 fatty acids as a dominant constituent of chia seed oils reduces the detrimental oxidative effects of myocardial infarction because of its potent antioxidant properties either by increasing the activity of antioxidant enzymes such as catalase, superoxide dismutase or by increasing the level of non-enzymatic antioxidant markers such as GSH [41].

The elevated ROS level also decreases the expression of the nuclear factor erythroid 2-related factor (Nrf2), which enhances cell susceptibility to more oxidative stress and apoptosis [42]. DOX has been reported to decrease Nrf2/HO-1 expression to induce oxidative stress-mediated injury [43]. Many studies have reported that chia seed oil modulates Nrf2/HO-1 pathway and thereby protects from DOX-induced oxidative injury [44–46]. In this study, the inhibitory effect of chia seed oil on oxidative stress was demonstrated by a decrease in MDA and an increase in GSH levels, which indicates its anticipated cardioprotective mechanism. The normalisation of serum CK, CK-MB and AST activities supports the defence of chia seed oil against heart injury through a potential membrane stabilising effect.

## Conclusion

The present study collectively demonstrated that chia seed oil could protect against DOX-induced cardiac toxicity in female Wistar rats by inhibiting oxidative stress. Our study opens the perspective to clinical studies to precisely considering chia seed oil as a potential chemoprotectant nutraceutical in the combination chemotherapy with DOX to limit its cardiotoxicity.

## Data Availability

All data are available and can be given as per request.

## Abbreviations

**DOX**: Doxorubicin
**CSO**: Chia seed oil
**CK-MB**: Creatine kinase-MB
**LDH**: Lactate dehydrogenase
**AST**: Aspartate aminotransferase
**CK**: Creatine kinase
**MDA**: Malondialdehyde
**GSH**: Reduced glutathione
**Nrf2**: Nuclear factor erythroid 2-related factor 2
**HO-1**: Heme oxygenase-1
**DMSO**: Dimethyl sulfoxide
**ROS**: Reactive oxygen species
**RNS**: Reactive nitrogen species
**H & E**: Haematoxylin and eosin
**ECG**: Electrocardiogram
**TBA**: Thiobarbituric acid
**TCA**: Trichloroacetic acid
**DTNB**: 5, 5′-Dithiobis (2-nitrobenzoic acid)
**PUFA**: Polyunsaturated fatty acids
**CPCSEA**: Committee for the purpose of control and supervision on experiments on animals
